# Increasing Hepatitis E Virus Seroprevalence in Domestic Pigs and Wild Boar in Bulgaria

**DOI:** 10.3390/ani10091521

**Published:** 2020-08-28

**Authors:** Katerina Takova, Tsvetoslav Koynarski, Ivan Minkov, Zdravka Ivanova, Valentina Toneva, Gergana Zahmanova

**Affiliations:** 1 Department of Plant Physiology and Molecular Biology, University of Plovdiv, 4000 Plovdiv, Bulgaria; katerina.takova@gmail.com (K.T.); toneva@plantgene.eu (V.T.); 2 Department of Animal Genetics, Faculty of Veterinary Medicine, Trakia University, 6000 Stara Zagora, Bulgaria; tkoynarski@gmail.com; 3 Institute of Molecular Biology and Biotechnologies, 4000 Plovdiv, Bulgaria; minkov@plantgene.eu (I.M.); mrkzbg@gmail.com (Z.I.); 4 Center of Plant Systems Biology and Biotechnology, 4000 Plovdiv, Bulgaria

**Keywords:** hepatitis E virus, zoonotic transmission, foodborne infection, prevalence, boar, pigs

## Abstract

**Simple Summary:**

Hepatitis E virus (HEV) is a lesser-known hepatitis virus, but its worldwide spread is undisputed and has increased in recent years. The zoonotic spread of HEV, mainly due to genotype (gt) 3, emerged in developed countries in the past decade. In addition, transmission via contaminated meat from pigs and boars was also established. Detailed analysis of viral dynamics and distribution is needed in order to identify associated risk factors. The aim of the current study is to present new and additional data on the HEV distribution among pigs, and for the first-time, also among the wild boar population in Bulgaria.

**Abstract:**

(1) Background: Hepatitis E virus (HEV) is a causative agent of acute viral hepatitis, predominantly transmitted by the fecal–oral route. In developed countries, HEV is considered to be an emerging pathogen since the number of autochthonous cases is rising. Hepatitis E is a viral disease with a proven zoonotic potential for some of its genotypes. The main viral reservoirs are domestic pigs and wild boar. Consumption of undercooked meat, as well as occupational exposure, are key factors for the spread of HEV. In order to evaluate the risks of future viral evolution, a detailed examination of the ecology and distribution of the virus is needed. The aim of the present study is to investigate the prevalence of anti-HEV IgG Ab in domestic pigs and wild boar in Bulgaria; (2) Methods: In this study, during the period of three years between 2017 and 2019, 433 serum samples from 19 different pig farms and 1 slaughterhouse were collected and analyzed. In addition, 32 samples from wild boar were also collected and analyzed during the 2018–2019 hunting season. All samples were analyzed by commercial indirect ELISA; (3) Results: Overall, HEV seroprevalence was 60% (95% CI 42.7–77.1) in domestic pigs and 12.5% (4/32) in wild boar. The observed seroprevalence of the slaughter-aged pigs was 73.65% (95% Cl 58.7–87.3). Prevalence in domestic pigs was significantly higher in the samples collected during 2019 (98% (95% Cl 96.1–99.9)) compared to those collected during 2017 (45.33% (95% CI 2.7–87.3)) and 2018 (38.46% (95% CI 29.1–49.7.); (4) Conclusions: Our findings suggest that domesticated pigs and wild boar might be the reason for the increased HEV transmission across Bulgaria. The genotypic characterization of HEV found in pigs, wild boar and humans will give a more accurate view of the zoonotic transmission of this virus.

## 1. Introduction

Hepatitis E virus (HEV) is one of the leading causes of acute hepatitis worldwide. The hepatitis E infection is usually self-limiting in healthy human patients. However, immunocompromised, co-infected, and transplanted individuals may develop a fulminant infection with a high risk of developing a chronic disease. In addition, pregnant women infected with HEV have shown a higher mortality rate of up to 30% compared to 0.1–4% observed in the general population [1]. 

HEV is a small icosahedral virus from the *Hepeviridae* family, which is comprised of two genera *Orthohepevirus* and *Piscihepevirus*. The genus *Orthohepevirus* contains four species designated as *Orthohepevirus* A through D. Within the species *Orthohepevirus* A, 8 distinct mammalian genotypes (gt) have been identified so far (HEV 1–8) [2,3]. Gt 1 and gt 2 infect only humans and lead to epidemic outbreaks in developing countries located in Africa and Asia, as well as in Mexico. The HEV gt 1 and gt 2 are transmitted mainly through the fecal–oral route due to fecal contamination of drinking water [4]. In contrast, gt 3 and gt 4 have proven zoonotic potential and infect humans and several other animal species, such as pigs, wild boar, and deer [5,6,7]. Zoonotic transmission of gt 3 or gt 4 occurs through consumption of undercooked meat or contact with infected animals [8]. Gt 5 and gt 6 infect wild boar, while gt 7 infects dromedary camels. A recent study showed zoonotic transmission of gt 7 related to the consumption of camel meat. Gt 8 infects the Bactrian camel [9]. 

In Europe, HEV gt 3 infection is increasingly being recognized as an emerging disease [10]. Domestic pigs and wild boar are considered as the main animal reservoirs of HEV gt3. The reported anti-HEV IgG seroprevalence in swine herds ranges between 30% and 100% [11,12,13,14,15,16]. In Norway [12], 90% of the studied herds and 73% of the individual serum samples tested positive for specific IgG antibodies. Similar results were observed in many of the other European countries as well. The data from Italy shows that 97.43% of the tested herds and 50.21% of the serum samples were also positive for HEV specific IgG antibodies [13]. In Spain, 97.65% of the herds and 41.9% of the serum samples were positive as well [14].

HEV transmission within a herd occurs naturally through direct contact with HEV infected animals or with excreta from sick animals, or through contaminated water or food sources [17]. Animals can contract the HEV virus at various times during their growth period. Most studies show that pigs became infected between the age of 8 and 15 weeks, and some of them remain positive at slaughter [18,19,20,21]. In addition, it was shown that 2–15% of pigs were infected with HEV at slaughter [22]. The HEV viremia usually lasts up to 2 weeks with viral excretion, through feces, occurring usually between weeks 3 and 7 [20]. The infection in pigs causes the development of microscopic lesions in the liver, with asymptomatic clinical presentation [23]. Hepatitis E antibody seroconversion in pigs occurs between week 11 and week 13, following the waning in maternal antibodies, where IgM anti-HEV antibodies peak first, followed by the IgG anti-HEV antibodies [21].

Recent studies in Europe revealed molecular evidence for HEV infection in wild boar and high HEV seroprevalences [24,25,26,27,28]. The HEV disease-dynamic in wild boar is similar to domestic pigs; the infection frequency also increases with age as well as the possibility of viral chronification [29]. Viral transmission to humans is observed due to consumption of undercooked meat. Occupational exposure is a risk factor; veterinarians, slaughterers, and forest workers have shown higher anti-HEV antibody prevalence in comparison to people with other occupations [30]. Therefore, knowledge of the viral presence and dynamic in different hosts is crucial to establish prevention and healthcare approaches and measures.

Studies on the distribution of HEV in humans across Bulgaria show that the most acute hepatitis E cases in the country are caused by the HEV-3 subtypes 3*e*, 3*f* and 3*c* [31,32]. The first immunological study of pigs in Bulgaria from 2017 reported over 75% seropositivity. A noncommercial ELISA test based on the HEV ORF2 110–610 capsid protein was used to detect anti-HEV IgG antibodies in domestic pigs [33]. Another study reports overall HEV seroprevalence of 60.3%. More specifically, in weaners—25%, in fattening pigs—75.8%, and in sows—80% [34]. To date, there is no available information about HEV seroprevalence in Bulgarian wild boar. This study aims to provide further knowledge on the distribution of HEV in pigs across Bulgaria and to broaden perspectives of mapping the spread. Moreover, it provides the first data ever on HEV prevalence in the wild boar population within the Bulgarian territory. 

## 2. Materials and Methods 

### 2.1. Sample Collection and Selection Criteria

**Figure 1:**
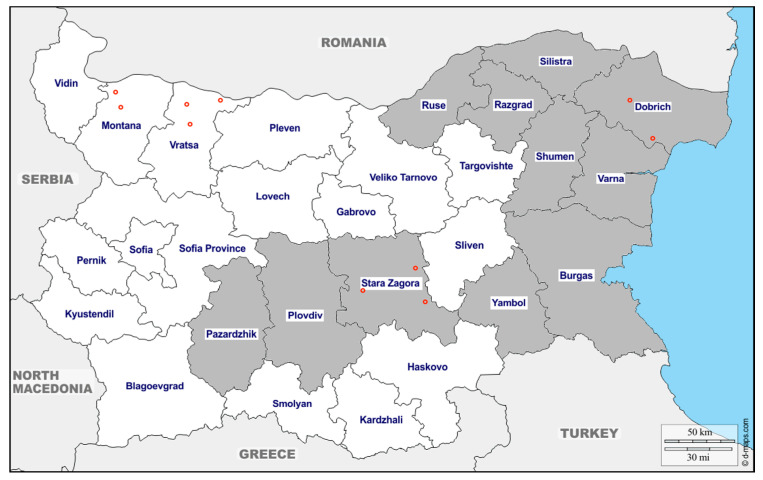
The map represents the origin of the farming pigs tested in grey. The origins of the wild boar’s testes are marked with red circles.

In total, 433 swine blood samples were collected from 19 different pig farms and 1 slaughterhouse, located in eleven districts across Bulgaria—Pazardzhik, Plovdiv, Stara Zagora, Yambol, Ruse, Razgrad, Silistra, Shumen, Varna, Dobrich, and Burgas district. The samples were collected during 2017, 2018, and 2019. Pig samples from 2017 (*n* = 75) were collected from two small pig farms (under 1000 pigs per farm) and a slaughterhouse, primarily used by local farmers. The age of swine tested was 5–6 months and older. Sera from 2018 (*n* = 158) were taken from industrial pig farms (over 1000 pigs per farm). Samples were taken from two age-groups, 6-month-old pigs that weighed around 80–100 kg (*n* = 78), and approximately 3-month-old pigs that weighed around 30 kg (*n* = 80). The 3-month-old pig samples were collected from four farms. Twenty samples were collected from each of the farms located in Shumen (2 farms), Dobrich and Burgas districts. Two of the farms (in Shumen and Dobrich districts) were also used for collection of 6-month-old pig samples. The 6-month-old pig samples were from five farms, located in four districts—Ruse (2 farms), Dobrich, Varna, and Shumen. The blood samples from 2019 (*n* = 200) were from ten farms from ten districts—Pazardzhik, Plovdiv, Stara Zagora, Yambol, Ruse, Razgrad, Silistra, Shumen, Varna and Dobrich (Figure 1). Twenty sera-samples from fattening farm pigs (5–6 months old) were collected from each farm. Information regarding their sex had not been recorded. The 6-month and the 3-month-old pig samples, collected during 2018, were chosen to examine the dynamics of the viral response through different pig ages. 

Geographically, districts Ruse, Razgrad, Silistra, Shumen, Dobrich and Varna are considered part of Northern Bulgaria. Districts Plovdiv, Stara Zagora, Pazardzhik, Yambol and Burgas are a part of Southern Bulgaria. The Stara Planina Mountain range located in the middle of the country, stretches from the eastern to the western borders of Bulgaria, acting as a natural divide, between Northern and Sothern Bulgaria.

In addition, 32 meat chunks from wild boar were collected for meat juice extraction. The samples were collected from animals hunted in Stara Zagora, Vratsa, Montana, and Dobrich districts during the official hunting season. Information regarding their sex and age was not recorded. 

Eight samples were collected in district Stara Zagora (1–3 village Dalboki; 4–5 village Topolyane; 6–8 village Sladak Kladenec). Ten samples were collected from district Vratsa (1–6 village Butan; 7–8 village Leskovec; 9–10 village Dobrolevo). From district Montana, six samples were collected (1–2 village Medkovec; 3–6 village Staliyska Mahala), and from Dobrich district, eight (1–4 village Kozloduyci; 5–8 village Prilep). The origin given on the map indicates the closest place to the hunted area (Figure 1). 

### 2.2. Ethics Statement 

The authors report that all animals were treated following ethical principles approved by Bulgarian authorities. Sera samples were collected during routine health screenings of the pigs. Meat chunk samples from wild boar were obtained from hunted animals during the 2018/2019 hunting season. This study did not involve purposeful killing of animals. 

### 2.3. Sample Processing 

Blood samples from pigs (*Sus scrofa domesticus*) were taken by puncture of the sinus ophthalmicus. The blood samples were kept in plain vacutainers (without anticoagulant reagent) at room temperature (20 °C) until visible clot retraction. After centrifugation at 1500 g for 10 min, the sera were separated and kept at −20 °C until use.

Meat chunks from wild boar (*Sus scrofa*) were squeezed and meat juice samples were extracted. Approximately 200 mg of the meat was homogenized with a ceramic bead for 30 s at 4 speed (MP Biomedicals, Irvine, CA, USA). Samples were then squeezed through a layer of Miracloth and kept at −20 °C until further use.

### 2.4. Testing Assay

A commercial ELISA (PrioCHECK^®^ HEV Ab porcine, Prionics AG, Schlieren, Switzerland) assay was used for the detection of HEV-specific antibodies. According to the manufacture protocol, dilutions of 1:100 for sera samples and 1:10 for meat juice samples were used. The test claims 91.0% sensitivity and 94.1% specificity. The cut-off value (Co) was calculated according to the manufacture protocol, as a means of optical density 450 (OD450) of the cut-off control multiplied by 1.2. 

### 2.5. Data and Statistical Analysis

HEV prevalence was estimated from the ratio of positive samples to the total number of samples analyzed, with binomial confidence intervals of 95%. We calculated HEV prevalence during 2017, 2018 and 2019 in order to evaluate changes over time. Variables were expressed as percentages. Frequencies were compared using the chi-square test, and significance was set at a two-tailed *p*-value of less than 0.05. All data analysis was performed by Numbers 2019 (Apple Inc., Cupertino, CA, USA), Excel 2007 (Microsoft, Redmond, WA, USA) and SPSS Statistics 19.0 (IBM Corp., Armonk, New York, NY, USA). 

## 3. Results

### 3.1. HEV Seroprevalence in Domestic Pigs

The overall HEV seroprevalence in domestic pigs was 60.05% (260 of 433 samples). There were no positive samples (0 of 80 samples) among the group of 3-month-old pigs (2018) originated from Shumen (2 farms), Dobrich (1 farm) and Burgas (1 farm) districts. Among the slaughter-aged pigs, the seroprevalence was 73.65% (260 of 353 samples). With respect to herd prevalence of anti-HEV antibodies, 17 out of 17 pig farms had HEV-positive pigs at slaughter age, demonstrating a 100% prevalence in the pig herds. The seropositivity in the slaughter-aged pigs varies based on the tested year and district (Table 1).

**Figure 2:**
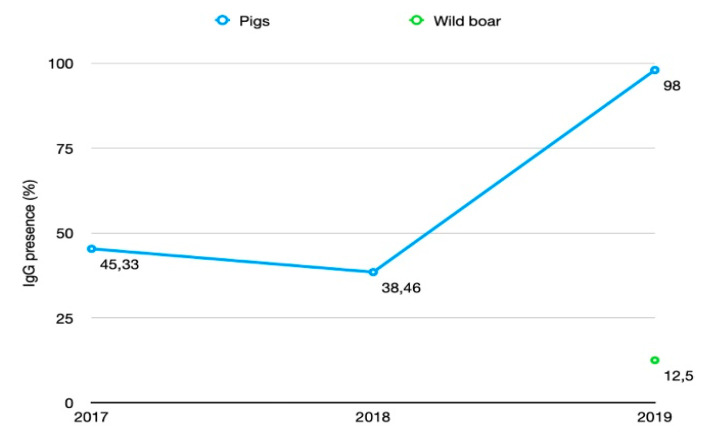
HEV IgG positive test results by year.

Samples collected during 2019 showed 98% (196 of 200 samples) seroprevalence. In 2017 and 2018, the HEV seroprevalence was 45.33% (34 of 75 samples) and 38.46% (30 of 78 samples) respectively (Figure 2). 

The observed high HEV seroprevalence during 2019 (98% (95% Cl 96.4–99.9)) compared to the previous two years, 2017 (45.33% (95% CI 2.7–87.3)) and 2018 (38.46% (95% CI 29.1–49.7)), is statistically significant, with a chi-square (χ2) value of 149.02 and a *p*-value <0.01 (Table 2).

**Figure 3:**
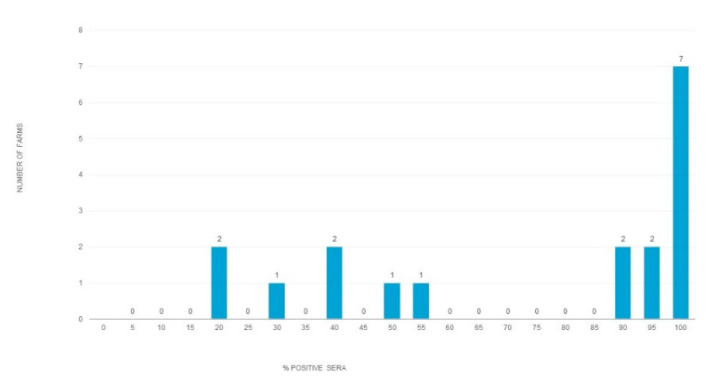
Variance between farms in HEV seroprevalence among slaughter-aged pigs. The table includes data collected from 17 farms and 1 slaughterhouse during the period of 2017–2020. The chart represents the number of farms (*Y*-axis) based on the percentage of positive sera per farm (*X*-axis).

HEV seroprevalence did not differ significantly between 2017 and 2018 (χ2 = 0.742, df 1, *p* > 0.01). The seroprevalence in 2019 (98% (95% CI 96.4–99.9)) was significantly higher than the seroprevalence in 2017 and 2018 (χ2 = 146.2139, df 1, *p* < 0.00001). The HEV seroprevalence varied between the farms from 20% to 100% (73.05, (95% CI 58.2–88)) (Figure 3). The presence of specific IgG differed between farms from the same region. For example, in 2018, one of the two farms in Ruse district showed 20% positivity, while the other 40%. In Stara Zagora district, the variation between farms was even greater. In 2017, one of the two studied farms showed 20% positivity while the other, 88%. 

HEV seroprevalence did not differ significantly between farms located in Southern and Northern Bulgaria (χ2 = 0.2997, and a *p*-value of 0.58) (Table 3).

### 3.2. HEV Seroprevalence in Wild Boar

Twelve and half percent, or 4 of the 32 samples from wild boar, collected from four different regions during the 2018/2019 hunting season (Figure 1), were positive for anti-HEV IgG antibodies (Figure 2). Three of them originated from Dobrich district (2 near village Kozloduyci and 1 near village Prilep) and one from Stara Zagora district, near village Sladak Kladenec.

## 4. Discussion

The proven zoonotic potential of HEV in pigs, combined with the relatively high prevalence of HEV positive pigs in Europe, [21] and the presence of HEV infection in slaughter-aged pigs [35], may have a negative impact on the safety of pork products. 

This research represents the first study on the HEV infection dynamics conducted during a three-year period in 19 different farms from eleven different Bulgarian districts. HEV seroprevalence was also shown in wild boar during the 2018/2019 hunting season. The overall seroprevalence obtained from domestic pigs was 60% (95% CI 42.7–77.1) and 12.5% (4/32) in wild boar. This rate is similar to the reported rate of HEV in pigs and wild boar in several European countries [15,16,29]. From the two studied age-groups of pigs, only the 6-month-old pigs were found to be seropositive. The 3-month-old pigs were not positive for anti-HEV IgG, confirming previous observations, showing that viremia most often occurs between the age of 2 and 4 months, and that anti-HEV IgG antibodies appear late during the period of viremia [36]. In addition, there are factors that can significantly delay HEV seroconversion, for example, co-infection with porcine reproductive and respiratory syndrome virus (PRRSV) and the presence of maternal antibodies. These factors may lead to HEV RNA presence at slaughter age, which appears to be a human health risk factor [37,38].

In this study, the HEV seroprevalence for anti-HEV IgG among the 6-month-old pigs, from different herds from ten Bulgarian districts (Table 1), ranged from 20% to 100%. The difference in seroprevalence suggests different transmission dynamics or exposure routes for HEV within the studied pig herds. A study from the Netherlands reported higher seropositivity in organic farms compared to conventional ones. The reason may be a greater contact frequency between pigs [39]. Other farming practices associated with HEV presence seem to include late weaning, mingling practices at the nursery stage, and poor hygiene measures [40]. 

The significantly higher seroprevalence observed in the sample cohort from 2019 compared to those from 2017 and 2018 suggests a rise in HEV infections in pigs, which may decrease or remain steadily high in the following years, which is concerning. Our findings complement the observations from previous studies done in Bulgaria and in other Balkan countries. Earlier studies on the distribution of HEV among Bulgarian pigs showed 40% overall seroprevalence (35/85) in pigs [41]. Another study showed 60.3% positive results for anti-HEV IgG in pigs from three Bulgarian districts [34]. A Romanian study showed 39% seropositivity among industrial pigs and 50% seroprevalence in backyard pigs [42,43]. In Serbia, a total of 315 serum samples from 3 to 4-month-old healthy backyard pigs were collected, of which 34.6% were positive for anti-HEV IgG [44]. 

A number of studies have shown that the overall seroprevalence in wild boar was significantly lower than the seroprevalence in domestic pigs [16,45,46]. Our study demonstrates that HEV is present among the wild boar population in Bulgaria, and it also confirms the observed lower seroprevalence among wild boar compared to domestic pigs. In our pilot investigation, only 12.5% of the wild boar samples were positive for HEV IgG antibodies. Differences seem to exist between the studied geographic areas (3 of the 4 positive samples were from one, Dobrich, of the four studied districts). However, it is difficult to draw meaningful conclusions with respect to the distribution of the virus due to the limited number of samples. Very similar results were reported in two studies across Romania, showing 10.29% and 9.61% HEV seroprevalence among the studied wild boar populations [47,48]. Interestingly, Dobrich is the only one of the four districts, where wild boar samples were collected, that shares a land border with Romania. Two studies, from France and the Netherlands, have also shown that HEV is endemic for these territories, with seropresence of 14% and 12%, respectively [49,50]. A research team from Spain reported a 23% presence of HEV RNA and suggested that the dynamics of the virus in wild boar may be seasonal, with the highest peak at the beginning of the hunting season, late October and November [26]. In addition, people with occupational exposure, such as hunters and forest workers, tend to show a higher presence of specific antibodies, which suggest an increased risk for HEV infection among them [30]. Although the researched pool is too small to be significant, this study is the first-ever proof for the spread of HEV among the wild boar population in Bulgaria. The results display the need for further studies to evaluate the degree of prevalence in this population and the associated risks of HEV prevalence in livers.

There are only a few immunological studies done in Bulgaria focused on HEV in humans. Two of them include only patients with clinical symptoms of acute hepatitis or liver failure. One of the records, collected between 2004 and 2012, showed 2.48% (20/806) presence of specific antibodies [51]. The other showed 13.2% (43/325) presence of anti-HEV IgM and 20.9% presence of anti-HEV IgG in patients studied between 2012 to 2016 [52]. Another report, from district Plovdiv, which includes 741 samples, collected between 2012 and 2013 from random patients, showed 9.04% presence of anti-HEV IgG and 1.48% of both anti-HEV IgG and IgM [53]. All of them showed a higher presence, which appears to increase with age. However, none of the studies investigated if there is a correlation between HEV in humans and pigs. In Bulgaria, two HEV phylogenetic studies confirmed the presence of HEV *Subtypes 3e*, *3f*, *3c*, *3i*, and *3hi*, and *Subtype 1*, in human samples [31,52]. Most of these subtypes refer to domestic pigs and wild boar, which reveals the probability of zoonotic transmission of HEV infection to humans. In addition, the higher frequency of detection of anti-HEV antibodies in veterinarians and pig farm workers strongly suggests the zoonotic potential of the disease [54,55]. Most likely, the transmission of the virus to the general public is predominantly a result of the consumption of undercooked pork and game meat [7,46,47].

This investigation has shown that domestic pigs and wild boar in Bulgaria are widely infected with HEV. There is a need for the production of HEV immunogenic proteins, which can be used for development of serological tests for detection of the HEV infection. In our previous study, we described a method for the production of recombinant HEV open reading frame (ORF) 2 protein in *Nicotiana benthamiana* plants and the successful use of this protein for serological detection of anti-HEV antibodies in both humans and swine [33,56]. Other studies have also demonstrated the efficacy of rORF2 gt 3 produced in various expression systems in the detection of anti-HEV serum Ab [57,58]. Ultimately, one of our main goals is to develop an HEV diagnostic kit based on the plant-derived rORF2 capsid protein. Such a kit would be cost-effective, and it would allow routine HEV testing, especially in developing countries.

## 5. Conclusions

For the first time, this study shows that HEV circulates in the Bulgarian wild boar population. The results of the HEV IgG seroprevalence in Bulgarian wild boar and domestic pigs further characterizes the presence of the Hepatitis E viral infection in Bulgaria, thus highlighting a significant concern for zoonotic virus transmission through consumption of raw or undercooked meat and direct contact with animals. Our results suggest that follow up studies, investigating the zoonotic transmission of this virus to humans, are needed, as well as measures for adequate meat product treatments, and thorough monitoring of the HEV disease distribution. 

## Figures and Tables

**Table 1:** Anti-HEV IgG among slaughter-aged pigs (6-month-old) from different Bulgarian districts during 2017, 2018, and 2019. N designates the number of total samples; *n*, the number of positive samples; the districts are listed in alphabetical order.

Year			Dobrich	Pazardzhik	Plovdiv	Razgrad	Ruse	Varna	Shumen	Silistra	Stara Zagora	Yambol	Total
2017	IgG+	*n*									34		34
		%									45.33		45.33
	Total	N									75		75
2018	IgG+	*n*	6				9	7	8				30
		%	33.33				30	46.67	53.33				38.46
	Total	N	18				30	15	15				78
2019	IgG+	*n*	19	20	20	18	20	20	20	20	20	19	196
		%	95	100	100	90	100	100	100	100	100	100	98
	Total	N	20	20	20	20	20	20	20	20	20	20	200
Total	IgG+	*n*	25	20	20	18	29	27	28	20	54	19	260
		%	65.79	100	100	90	58	77.14	80	100	56.84	95	73.65
	Total	N	38	20	20	20	50	35	35	20	95	20	353

**Table 2:** Time factor of the HEV seroprevalence. N denotes the number of animals; OR is the odds ratio; 95% CI confidence interval; *p*—*p*-value and in the brackets [the chi-square statistic for each cell].

Variable	N	HEV Positive	HEV Negative	OR (95% CI)	*p*
2017	75	34 (55.67) [8.43]	41 (19.33) [24.28]	1.3 (95% CI: 0.69–2.5)	0.3894
2018	78	30 (57.89) [13.44]	48 (20.11) [38.69]	0.75 (95% CI: 0.39–1.43)	0.3894
2019	200	198 (148.44) [16.55]	2 (51.56) [47.64]	1.8984 (95% CI: 1.22–2.94)	0.0044

**Table 3:** The regional factor affecting HEV seroprevalence. N denotes the number of animals, *p*—*p*-value and in the brackets [the chi-square statistic for each cell].

Region	Positive HEV IgG	Negative HEV IgG	Totals	*p*
Southern Bulgaria	113 (110.88) [0.04]	32 (34.12) [0.13]	145	0.58
Northern Bulgaria	147 (149.12) [0.03]	48 (45.88) [0.1]	195	0.58
